# Academic stress and internet gaming addiction in college students: a moderated mediation model with anxiety as mediator and peer support as moderator

**DOI:** 10.3389/fpsyt.2026.1958057

**Published:** 2026-10-15

**Authors:** Jingjing Li, Bao Liu, Yu Wang

**Affiliations:** 1 School of Health Management, Guangzhou Medical University, Guangzhou, China; 2 Student Affairs Department, North China Institute of Aerospace Engineering, Langfang, China; 3 Research Center of Rural Education and Cultural Development/School of Education, Hubei University of Science and Technology, Xianning, China

**Keywords:** academic stress, anxiety, college students, internet gaming addiction, peer support

## Abstract

**Background:**

Academic stress is a known risk factor for internet gaming addiction among college students; however, the underlying mechanisms remain not fully elucidated. The current study aimed to examine the mediating role of anxiety in the relationship between academic stress and internet gaming addiction, as well as the moderating role of peer support.

**Methods:**

A cross-sectional survey was conducted among 636 Chinese college students in China. Participants completed measures of academic stress, anxiety, peer support, and internet gaming addiction. Analyses were performed using the PROCESS macro for SPSS. Model 4 was used to test mediation, and Model 59 was used to test moderated mediation, with age and gender controlled for in all analyses.

**Results:**

Academic stress was positively associated with internet gaming addiction among college students (*r* = 0.28, *p* < 0.001), and anxiety mediated this relationship (indirect effect = 0.22, 95% CI = [0.16, 0.27]). Peer support significantly moderated this indirect effect by buffering the path from academic stress to anxiety (*b* = -0.18, *p* <.01). Specifically, for college students with lower peer support, the relationship between academic stress and anxiety was significant (*b* = 0.42, *p* <.001); for those with higher peer support, the relationship remained significant but weaker (*b* = 0.24, *p* <.001).

**Conclusion:**

Academic stress was associated with internet gaming addiction among college students through increased anxiety, and peer support served as a protective buffer in this mechanism by weakening the association between academic stress and anxiety. These findings highlight the importance of anxiety management and peer support interventions for preventing internet gaming addiction among college students facing academic stress.

## 1 Introduction

### 1.1 Research background and problem statement

According to the 57th Statistical Report on Internet Development in China released by the China Internet Network Information Center, as of December 2025, the number of internet users in China reached 1.125 billion, among which internet game users numbered 573 million, accounting for 50.9% of the total internet users (1). Internet games have a dual nature: on the one hand, they can enrich life experiences; on the other hand, they also carry the risk of addiction. Previous studies have shown that the prevalence of internet gaming addiction among Chinese college students is approximately 7.5% (2). Internet gaming addiction is defined as the persistent and recurrent use of the Internet to engage in games, often involving interaction with other players, leading to clinically significant impairment or distress (3). Previous studies have shown that internet gaming addiction among college students is associated with psychological problems such as anxiety, depression, and loneliness (4–6), and is also related to risky behaviors including non-suicidal self-injury and suicide (7–9). Given the high prevalence and serious consequences of internet gaming addiction among college students, identifying risk and protective factors is critical for developing effective prevention and intervention strategies.

### 1.2 Academic stress and internet gaming addiction

Previous studies have shown that academic stress is significantly associated with increases in both internalizing and externalizing problems among college students (10, 11). According to the general strain theory (12), academic stress constitutes a salient strain that elicits negative affective states. To alleviate these aversive emotions, individuals are motivated to engage in maladaptive coping behaviors that provide immediate escape from academic stress. Internet gaming, characterized by its immersive escapism, may serve as such a compensatory strategy, thereby elevating the risk of addiction among college students. Prior research has documented a significant association between academic stress and internet gaming addiction among college students (13, 14). For example, Gu and Mao (13) found that academic stress increased the tendency toward internet gaming addiction among college students through the mediating role of escape and coping motives. Putri et al. (14) found that both academic stress and nomophobia were significantly correlated with the intensity of internet gaming in a sample of 304 college students. Therefore, we propose the following hypothesis:

Hypothesis 1: Academic stress is positively associated with internet gaming addiction among college students.

### 1.3 Anxiety as a potential mediator

According to stress and coping theory (15), academic stress, as an external stressor, initiates individuals’ cognitive appraisal process and elicits negative emotions such as anxiety. When college students perceive insufficient coping efficacy, they are more likely to adopt internet gaming as an avoidant emotion-focused coping strategy to obtain immediate emotional relief by temporarily disengaging from real-life stressors, thereby increasing susceptibility to internet gaming addiction. Specifically, first, academic stress may trigger anxiety among college students. Wang et al. (16) found in a sample of 1,623 college students that academic stress positively predicted anxiety; Ouyang et al. (17) found in a sample of 959 college students that academic stress was positively correlated with academic anxiety. Second, college students with anxiety are more likely to develop internet gaming addiction. Chen et al. (5) found in a sample of 605 college students that anxiety was positively correlated with internet gaming addiction; Sobih et al. (18) found that social anxiety (as an important form of anxiety) was positively correlated with internet gaming addiction among college students.

In addition, previous studies have indicated that anxiety mediates the relationship between stress and maladaptive behaviors among college students (19–21). For example, in a sample of 488 college students, Jiang et al. (21) found that anxiety mediated the relationship between stress and aggressive behavior. Similarly, Chai et al. (19), with a sample of 1,672 college students, found that anxiety mediated the relationship between stress and unhealthy eating behaviors. Therefore, we propose the following hypothesis:

Hypothesis 2: Anxiety mediates the relationship between academic stress and internet gaming addiction.

### 1.4 Peer support as a moderator

Bronfenbrenner’s (22) ecological systems theory identifies family, school, and peers as important microsystems that shape individual development. Prior research has demonstrated that positive peer relationships serve as important protective factors against internet gaming addiction among college students (23, 24). For instance, Liu et al. (23), with a sample of 2,533 male college students, found that family support and peer support were protective factors against internet gaming addiction. Similarly, Teng et al. (24) conducted a longitudinal study with 1,054 college students and found that peer attachment negatively predicted subsequent internet gaming addiction.

According to ecological systems theory (22), peer support, as a key protective resource within the microsystem, can moderate through buffering mechanisms the relationship between risk factors and maladaptive outcomes among college students. Specifically, when facing academic stress, individuals with high peer support may receive emotional and instrumental assistance, thereby reducing reliance on maladaptive coping strategies such as internet gaming. Conversely, those with low peer support are more susceptible to stress-induced negative emotions and may turn to internet gaming as a maladaptive coping strategy. Therefore, peer support may moderate both the direct effect of academic stress on internet gaming addiction and the indirect effect through anxiety. Empirical research provides support for this theoretical view. For example, Zhou et al. (25), using a sample of 837 college students, found that psychological capital and peer support jointly moderated the relationship between cyberbullying victimization and depression—specifically, when college students had high levels of both psychological capital and peer support, the relationship was no longer significant. In addition, using a sample of 456 Chinese international students, Xu and Chai (26) found that peer support buffers the detrimental effects of academic pressure by enhancing students’ adaptive and integrative capacities. Therefore, we propose the following hypothesis:

Hypothesis 3: Peer support moderates the direct and indirect effects of academic stress on internet gaming addiction through anxiety.

### 1.5 Theoretical integration

Given that no single theory can fully explain the process through which academic stress leads to internet gaming addiction — general strain theory focuses on how stressors trigger problem behavior, stress and coping theory focuses on how individuals appraise and cope with stress, and ecological systems theory focuses on how contextual factors shape this process — the three theories differ in their explanatory emphases. This study integrates general strain theory (12), stress and coping theory (15), and ecological systems theory (22) to propose an integrative framework for examining how academic stress increases the risk of internet gaming addiction. Specifically, general strain theory provides the basis for why academic stress may elevate addiction risk; stress and coping theory further clarifies the psychological pathway, explaining how anxiety mediates the relationship between academic stress and maladaptive coping behaviors (such as internet gaming addiction); and ecological systems theory complements the two by identifying the boundary conditions under which this mediating process is attenuated (i.e., the buffering effect of peer support). Together, the three theories form a complementary causal chain — “stress source (general strain theory) → psychological mechanism (stress and coping theory) → contextual buffer (ecological systems theory)”. By jointly specifying the internal psychological mechanism (anxiety as the mediator) and the contextual boundary condition (peer support as the moderator), this integrated framework overcomes the limitations of single-theory perspectives and comprehensively illustrates how academic stress contributes to internet gaming addiction among college students within specific social contexts.

### 1.6 The present study

Although the associations among academic stress, anxiety, and internet gaming addiction have been well established, it remains unclear whether the mediating role of anxiety is moderated by social contextual resources such as peer support. Specifically, previous studies have mainly examined peer support as a main-effect protective factor (23), and few have tested whether it moderates the mediating process of academic stress → anxiety → internet gaming addiction.

Based on general strain theory (12), stress and coping theory (15), and ecological systems theory (22), this study examined (a) the association between academic stress and internet gaming addiction among college students, (b) the mediating role of anxiety in this association, and (c) the moderating role of peer support. The theoretical model proposed in this study is displayed in **Figure 1**.

**Figure 1:**
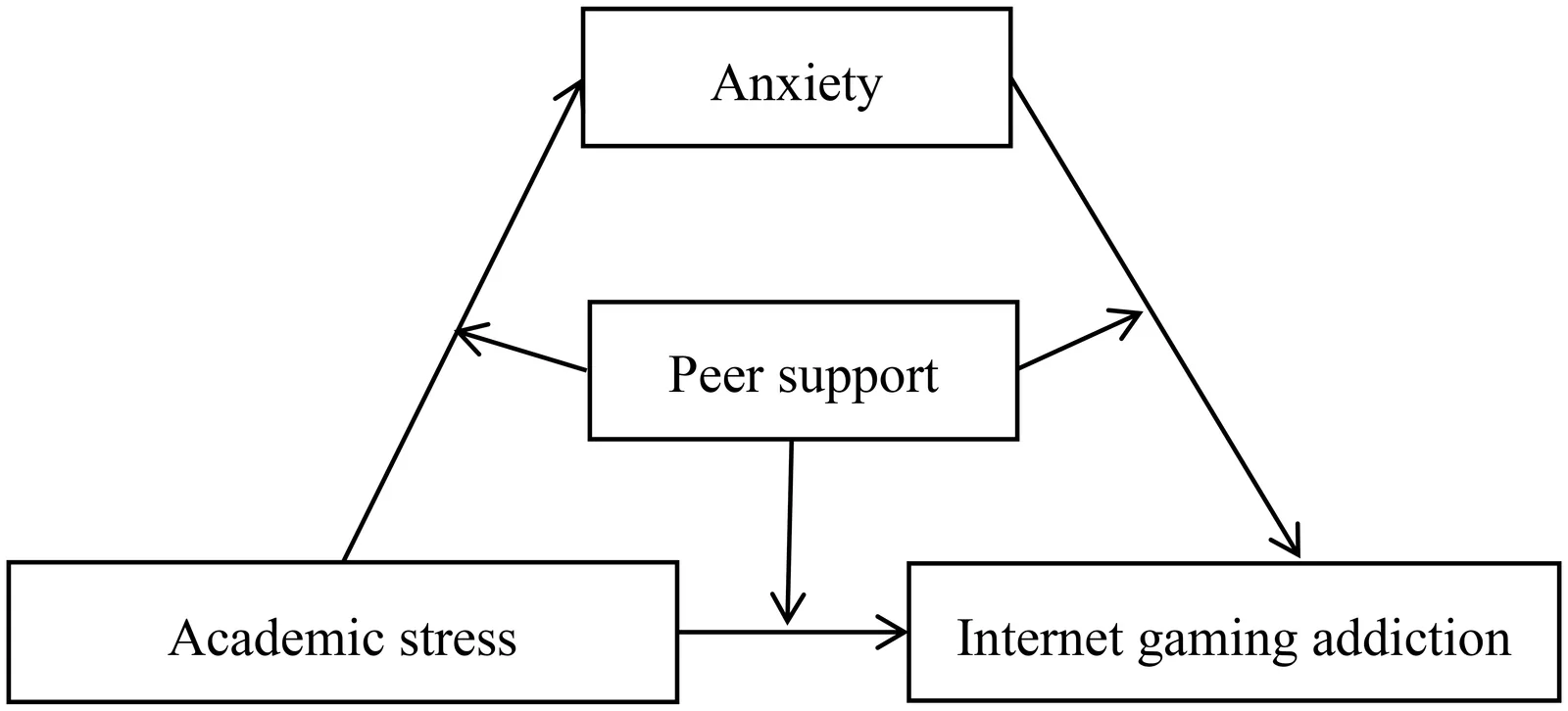
The proposed moderated mediation model.

## 2 Methods

### 2.1 Participants

This study adopted a cross-sectional research design. A total of 636 college students were recruited from a public comprehensive university in Xianning, Hubei Province, China. This study employed convenience sampling, with intact classes as the unit of group administration. We conducted group-administered surveys with all students in classes taught by the researcher. Classes were included because they were taught by the researcher and therefore readily accessible, rather than being randomly selected from a defined sampling frame. No stratification by university, college, grade level, or major was applied during sampling; grade-level information was collected through the questionnaire to characterize the sample composition.

Inclusion criteria: (a) Full-time undergraduate students enrolled in Chinese universities; (b) Present on the day of survey administration and voluntarily participated; (c) Provided written informed consent. Exclusion criteria: (a) Part-time students (e.g., continuing education students); (b) Students absent due to leave, suspension, or transfer on the survey day; (c) Those who did not provide consent or withdrew consent during the survey; (d) Senior-year students, as they were off campus for internships during the data collection period and therefore could not be reached for survey administration. Of these, 223 (35.1%) were male and 413 (64.9%) were female. In terms of grade level, 298 (46.6%) were freshmen, 194 (30.5%) were sophomores, and 144 (22.6%) were juniors. With respect to hometown type, 439 (69.0%) came from rural areas, 111 (17.5%) from towns, and 86 (13.5%) from cities. The age of the participants ranged from 18 to 23 years, with a mean of 19.76 years (SD = 1.19). A total of 636 valid questionnaires were obtained, yielding a valid response rate of 92.0%.

### 2.2 Procedure

This study obtained ethical approval from the Academic Ethics Review Committee of the university with which the author is affiliated. All participating college students provided informed consent prior to formal questionnaire administration. They were informed that the survey data would be collected anonymously through an online platform, used solely for academic research purposes, and that all personal information would be kept strictly confidential. Participants were also assured of their right to withdraw from the survey at any time without facing any adverse consequences.

The data collection procedure was conducted during class meetings (approximately 15 minutes). The researcher first explained the study purpose and procedures to the students, then projected a QR code on the classroom screen. Students scanned the code using their smartphones to access the online questionnaire (Wenjuanxing), where they completed the informed consent form before proceeding to the formal survey. The researcher remained in the classroom throughout to monitor the process and ensure independent completion.

The following quality control measures were implemented during data collection: (1) Missing data prevention: All items on the online platform were set as mandatory, requiring participants to answer all questions before submission; (2) Real-time monitoring: The researcher remained present in the classroom throughout to prevent discussion among students and ensure that each student completed the questionnaire independently; (3) Post-collection screening: after data collection, questionnaires with abnormally short completion times (less than 5 minutes, i.e., less than one-third of the normal completion time) or obvious patterned responding (e.g., consecutive identical options) were excluded.

### 2.3 Measures

#### 2.3.1 Academic stress

Academic stress was measured using the Academic Stress subscale of College Students Mental Health Screening Scale (27). This scale consists of three screening levels and 22 dimensions. The Academic Stress subscale belongs to the third-level screening. This subscale consists of 4 items (e.g., “I have put in great effort but still cannot keep up with my study progress”). Each item was rated on a 4-point Likert scale ranging from 1 (not at all like me) to 4 (very much like me). Mean scores were calculated for analysis, with higher scores indicating a higher level of academic stress. The validation study (27) reported that the internal consistency coefficient of the Academic Stress subscale was 0.61, and the test-retest reliability was 0.75, and the subscale was significantly positively correlated with the SCL-90 total score (r = 0.25, p <.001). Cronbach’s alpha in this study was 0.86.

#### 2.3.2 Anxiety

Anxiety was measured using the Chinese version of the anxiety subscale of the 21-item Depression Anxiety Stress Scales. The Chinese version was validated by Gong et al. (28), and the original scale was developed by Lovibond and Lovibond (29). This subscale comprises 7 items (e.g., “I felt scared without any good reason”). Each item is rated on a 4-point Likert scale ranging from 0 (does not apply to me) to 3 (applies to me very much). Mean scores were computed for analysis, with higher scores indicating higher levels of anxiety. The validation study (28) reported that the internal consistency coefficient of the anxiety subscale of the Chinese version of the DASS-21 was 0.79. The anxiety subscale was also significantly correlated with the State Anxiety Inventory (r = 0.531) and the Trait Anxiety Inventory (r = 0.552, p <.001). Cronbach’s alpha in this study was 0.91.

#### 2.3.3 Peer support

Peer support was measured using the Student-student support subscale of the Perceived School Climate Scale (30). This subscale consists of 13 items (e.g., “Students care about one another”). Each item was rated on a 4-point Likert scale ranging from 1 (never) to 4 (always). Mean scores were calculated for analysis, with higher scores indicating a higher level of peer support. The validation study by Jia et al. (30) reported good internal consistency for this subscale, with Cronbach’s α = 0.86 in the Chinese sample and α = 0.82 in the U.S. sample. In addition, the subscale was significantly negatively associated with depressive symptoms and positively associated with self-esteem in both samples, supporting its criterion-related validity. Although this scale was originally developed for adolescent populations, its three dimensions—teacher support, student–student support, and opportunities for autonomy—are also developmentally appropriate for college students. Previous research has applied this scale to Chinese college student samples and reported satisfactory reliability (e.g., 31, Cronbach’s alpha = .886). Cronbach’s alpha in this study was 0.91.

#### 2.3.4 Internet gaming addiction

Internet gaming addiction was assessed using the Internet Gaming Disorder Scale-Short-Form (IGDS9-SF) developed by Pontes and Griffiths (32). The measure comprises 9 items, with an example being “Do you feel excessively preoccupied with internet games, such that you continue to think about in-game scenes after logging off or plan your next gaming session?”. Each item was rated on a 5-point Likert scale ranging from 1 (never) to 5 (very often). Mean scores were calculated for analysis, with higher scores indicating a higher level of Internet gaming addiction. The validation study (32) reported that the IGDS9-SF demonstrated good internal consistency (Cronbach’s α = 0.87 for the overall sample) and supported a single-factor structure. The scale also showed good criterion-related and concurrent validity, as evidenced by significant correlations with weekly gameplay time (r = 0.319, p <.0001) and the IGD-20 Test (r = 0.816, p <.0001). Cronbach’s alpha in this study was 0.96.

### 2.4 Statistical analyses

To analyze the data, descriptive statistics were generated using SPSS 27.0. Pearson correlation analysis was used to examine the relationships among the main study variables. Two-tailed tests were used, and statistical significance was set at α = .05. Model 4 of the PROCESS macro (version 4.2) was used to examine whether anxiety mediated the relationship between academic stress and internet gaming addiction. Furthermore, Model 59 of the PROCESS macro (version 4.2) was employed to examine the moderating effect of peer support on both the direct and indirect associations between academic stress and internet gaming addiction. Lastly, gender and age were controlled for as covariates in the statistical models. Gender was dummy-coded as 1 = male and 0 = female. Gender and age were included as covariates in both Model 4 and Model 59. Specifically, they were entered into the covariates box in the PROCESS macro, meaning that they were controlled for all analytical paths, including the direct paths and the indirect effects. The same covariate inclusion strategy was applied consistently across both models.

For the mediation (Model 4) and moderated mediation (Model 59) analyses using the PROCESS macro (version 4.2), percentile bootstrap confidence intervals were employed with 5,000 bootstrap resamples. Statistical significance was set at α = .05 (two-tailed). An indirect effect was considered significant if the 95% percentile bootstrap confidence interval did not include zero.

Before the regression analysis, multicollinearity was assessed using the variance inflation factor (VIF) and tolerance via SPSS linear regression for all independent variables included in the models. The results showed that the VIF values ranged from 1.02 to 1.44, all below the commonly used cutoff of 5, and tolerance values ranged from 0.69 to 0.98, all above 0.10, indicating no serious multicollinearity.

Harman’s single-factor test was conducted to examine whether common method bias posed a threat in this study. The first common factor accounted for 34.41% of the total variance, which was below the 40% cutoff criterion, indicating that common method bias was not a serious concern.

## 3 Results

### 3.1 Preliminary analyses

**Table 1** presents the descriptive statistics and Pearson correlation coefficients for all variables. Normality was assessed for the four main study variables using skewness and kurtosis. The absolute values of skewness ranged from 0.04 to 1.03, and the absolute values of kurtosis ranged from 0.06 to 1.18, both within the acceptable ranges (skewness < 2, kurtosis < 7), indicating that the variables were approximately normally distributed. Although the Kolmogorov-Smirnov test was significant (*p* <.001), this test is highly sensitive to large sample sizes. Therefore, skewness and kurtosis were used as the primary criteria for evaluating normality, and Pearson correlation analysis was considered appropriate. Academic stress was significantly and positively related to both internet gaming addiction and anxiety. Additionally, anxiety was significantly and positively related to internet gaming addiction, whereas peer support was significantly and negatively related to internet gaming addiction.

**Table 1:** Descriptive statistics and correlations for all variables.

Variable	1	2	3	4	5	6
1. Gender	1.00					
2. Age	-0.10**	1.00				
3. Academic stress	-0.06	-0.07	1.00			
4. Anxiety	0.11**	-0.09*	0.39***	1.00		
5. Peer support	-0.21***	0.03	-0.11**	-0.43***	1.00	
6. IGA	0.32***	-0.09*	0.28***	0.51***	-0.43***	1.00
Mean	0.35	19.76	2.44	1.63	3.18	1.99
SD	0.48	1.19	0.66	0.62	0.51	0.85

Gender was dummy coded as 1 = male, 0 = female. IGA = internet gaming addiction. *p <.05. **p <.01. ***p <.001.

### 3.2 Mediation effect of anxiety

As shown in **Figure 2**, after controlling for age and gender, the regression model predicting anxiety was significant (*F* = 43.45, *p* <.001, *R²* = 0.17), and academic stress was significantly positively associated with anxiety (*b* = 0.37, *SE* = 0.03, *β* = 0.39, *p* <.001). Gender was significantly positively associated with anxiety (*b* = 0.17, *SE* = 0.05, *β* = 0.28, *p* <.001), whereas age was not significantly associated with anxiety (*b* = -0.02, *SE* = 0.02, *β* = -0.04, *p* >.05). The regression model predicting internet gaming addiction was also significant (*F* = 83.39, *p* <.001, *R²* = 0.35), anxiety was significantly positively associated with internet gaming addiction (*b* = 0.58, *SE* = 0.05, *β* = 0.43, *p* <.001), and academic stress was significantly positively associated with internet gaming addiction (*b* = 0.17, *SE* = 0.05, *β* = 0.13, *p* <.001). Gender was significantly positively associated with internet gaming addiction (*b* = 0.50, *SE* = 0.06, *β* = 0.59, *p* <.001), whereas age was not significantly associated with internet gaming addiction (*b* = -0.01, *SE* = 0.02, *β* = -0.01, *p* >.05). Extending this, bootstrap analyses with bias-corrected percentiles further substantiated the indirect pathway, showing that anxiety significantly mediated the relationship between academic stress and internet gaming addiction (indirect effect = 0.22, *SE* = 0.03, 95% CI = [0.16, 0.27]), with a completely standardized effect of 0.17.

**Figure 2:**
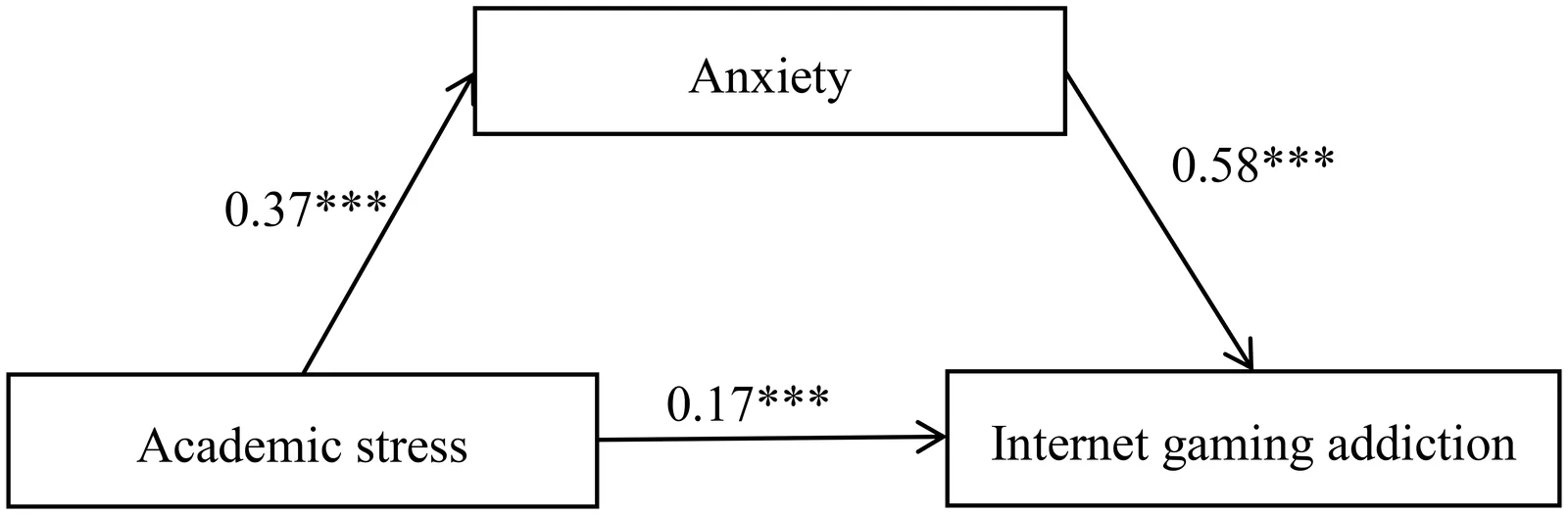
Model of the mediating role of anxiety in the association between academic stress and internet gaming addiction. ***p <.001. Values are unstandardized coefficients (b).

The total effect of academic stress on internet gaming addiction was 0.382. The direct effect was 0.167, accounting for 43.7% of the total effect, while the indirect effect through anxiety was 0.215, accounting for 56.3% of the total effect. These results indicate that anxiety partially mediated the relationship between academic stress and internet gaming addiction.

### 3.3 Moderated mediation

**Figure 3** displays the outcomes of the moderated mediation model. After controlling for age and gender, the regression model predicting anxiety was significant (*F* = 58.60, *p* <.001, *R²* = 0.32), and the regression model predicting internet gaming addiction was also significant (*F* = 55.61, *p* <.001, *R²* = 0.38). Gender (*b* = 0.05, *SE* = 0.04, *β* = 0.08, *p* >.05) and age (*b* = -0.03, *SE* = 0.02, *β* = -0.05, *p* >.05) were not significantly associated with anxiety. Gender was significantly positively associated with internet gaming addiction (*b* = 0.44, *SE* = 0.06, *β* = 0.53, *p* <.001), whereas age was not significantly associated with internet gaming addiction (*b* = -0.01, *SE* = 0.02, *β* = -0.01, *p* >.05). Peer support moderated the indirect effect of academic stress on internet gaming addiction via anxiety. Specifically, peer support significantly moderated the path from academic stress to anxiety (*b* = -0.18, *SE* = 0.06, *β* = -0.10, *p* <.01, 95% CI [-0.32, -0.03]). However, peer support did not significantly moderate the path from anxiety to internet gaming addiction (*b* = -0.01, *SE* = 0.10, *β* = -0.01, *p* >.05, 95% CI [-0.26, 0.25]), nor the direct path from academic stress to internet gaming addiction (*b* = 0.08, *SE* = 0.08, *β* = 0.03, *p* >.05, 95% CI [-0.12, 0.27]).

**Figure 3:**
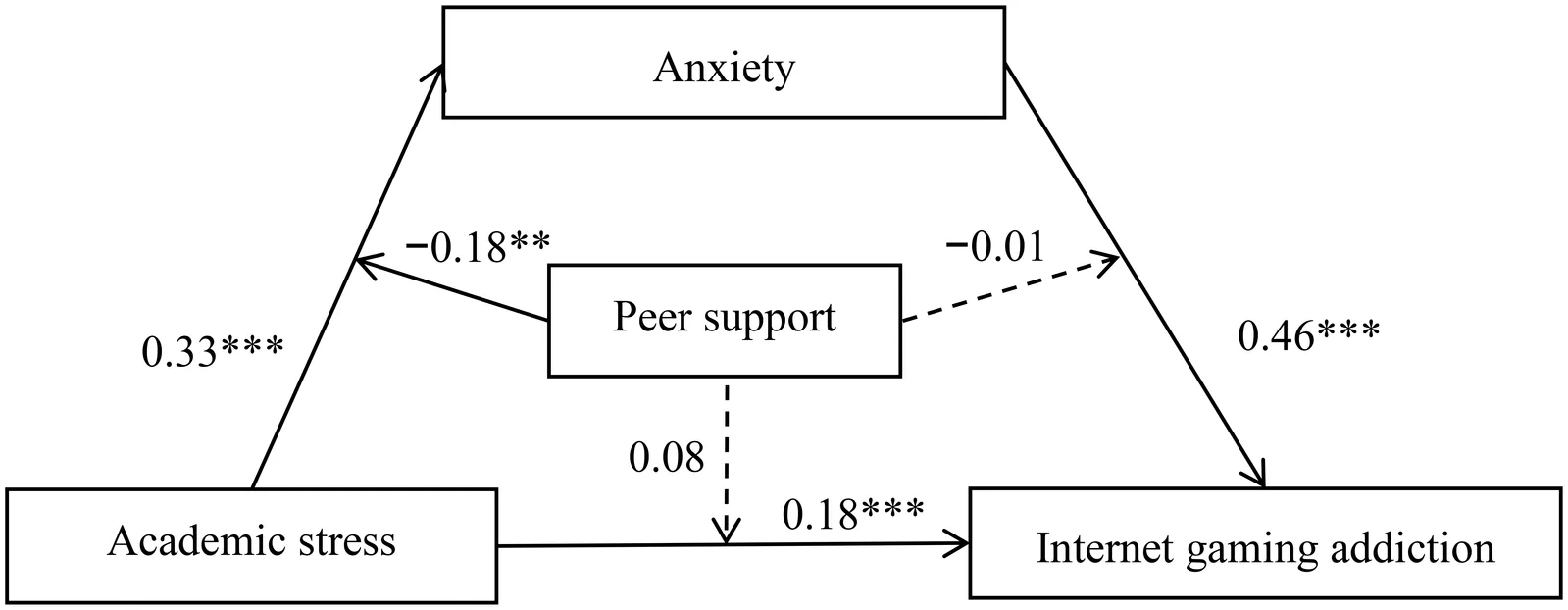
The moderating role of peer support on the indirect relationship between academic stress and internet gaming addiction. **p <.01. ***p <.001. Values are unstandardized coefficients (b).

To better understand how peer support moderates the indirect effect, we carried out simple slope tests. High and low peer support were operationalized as one standard deviation above and below the mean, respectively. As depicted in **Figure 4**, when college students reported lower peer support (-1 SD), the relationship between academic stress and anxiety was significant (*b* = 0.42, *SE* = .04, *p* <.001, 95% CI [0.33, 0.50]). However, when college students reported higher peer support (+1 SD), the relationship remained significant but weaker (*b* = 0.24, *SE* = .04, *p* <.001, 95% CI [0.15, 0.32]).

**Figure 4:**
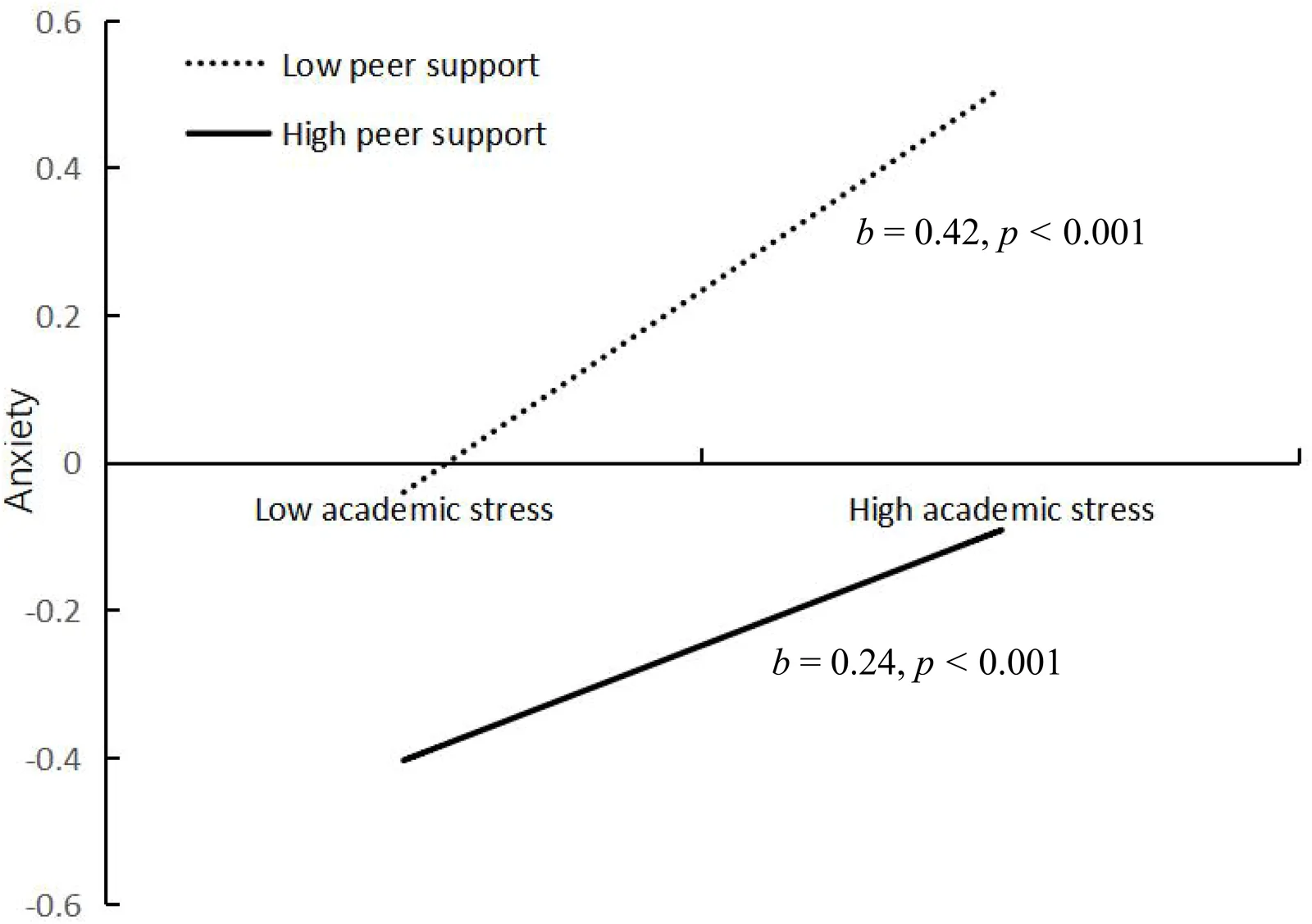
Interactive effect of academic stress and peer support on anxiety.

To quantify the conditional effects, we calculated the proportion of direct and indirect effects at different levels of peer support. At low peer support (-1 SD), the total effect of academic stress on internet gaming addiction was 0.331, with the direct effect accounting for 41.2% and the indirect effect through anxiety accounting for 58.8% of the total effect. At high peer support (+1 SD), the total effect was 0.325, with the direct effect accounting for 67.0% and the indirect effect accounting for 33.0% of the total effect. These results indicate that peer support buffers the mediating role of anxiety by weakening the first stage of the indirect pathway (academic stress → anxiety), thereby reducing the proportion of the indirect effect from 58.8% to 33.0%.

We conducted a *post-hoc* statistical power analysis. Since the significant effect was found only on the first stage of the mediation path, the actual supported conditional mediation structure corresponds to a first-stage moderated mediation model; we thus estimated power based on the Model 7 structure. This analysis assumes independent observations; the potential design effect associated with intact-class sampling is acknowledged as a limitation below. With N = 636 and *α* = .05, the achieved power was 1.00 for the conditional indirect effect, 0.98 for the direct effect, and 0.84 for the moderation effect on the X → M path. All values exceeded the conventional threshold of 1 − *β* ≥.80.

## 4 Discussion

Based on general strain theory (12), stress and coping theory (15), and ecological systems theory (22), this study examined the relationship between academic stress and internet gaming addiction among college students. The results indicated a significant positive correlation between academic stress and internet gaming addiction. Anxiety mediated this association, and peer support moderated the indirect pathway.

### 4.1 Academic stress and internet gaming addiction

Consistent with Hypothesis 1, academic stress showed a significant positive correlation with college students’ internet gaming addiction. This finding is consistent with previous studies (13, 14), further supporting that academic stress is an important risk factor for internet gaming addiction. Based on General Strain Theory (12), academic stress, as a stressor, may trigger negative emotions (16). When effective coping resources are lacking, college students may resort to online games to escape from real-life stress, which may in turn increase the risk of addiction.

### 4.2 The mediating role of anxiety

Consistent with Hypothesis 2, the present study found that anxiety significantly mediated the relationship between academic stress and internet gaming addiction among college students, highlighting anxiety as a key intermediary and illustrating the indirect pathway linking academic stress to internet gaming addiction via anxiety. This finding aligns with previous studies (19–21), further substantiating the mediating role of anxiety in the relationship between stress and maladaptive behaviors. Furthermore, this finding is in line with the stress and coping perspective (15). Specifically, academic stress triggers negative emotions such as anxiety in college students; to cope with anxiety, they may turn to internet gaming as a means of emotion regulation, thereby increasing the risk of addiction.

### 4.3 The moderating role of peer support

Partially consistent with Hypothesis 3, peer support significantly moderated the first stage of the mediating pathway (academic stress → anxiety), but did not significantly moderate the direct effect (academic stress → internet gaming addiction) or the second stage of the mediating pathway (anxiety → internet gaming addiction). When peer support was low, the relationship between academic stress and anxiety was stronger; when peer support was high, this relationship remained significant but was considerably weakened. This suggests that peer support buffered the association between academic stress and anxiety.

However, the moderating effect on the pathway from anxiety to internet gaming addiction was non-significant, indicating that the buffering role of peer support is stage-specific. As a social protective resource, peer support can effectively alleviate individuals’ negative cognitive appraisal of academic stress, thereby reducing the development of anxiety. Nevertheless, once anxiety is aroused, this negative emotion drives individuals to develop a tendency to escape reality through internet gaming, and this tendency is less susceptible to the buffering effect of peer support.

The present study reveals that peer support indirectly influences internet gaming addiction by moderating the relationship between academic stress and anxiety, thereby enriching the understanding of the pathways linking academic stress to internet gaming addiction among college students. This finding is consistent with previous studies (25, 26) as well as with ecological systems theory (22), namely, that peer support, as a key protective resource in the microsystem, can buffer the relationship between risk factors and maladaptive outcomes.

### 4.4 Theoretical explanation from an integrated perspective

Taken together, these three findings are not isolated from one another and jointly form an integrated theoretical account of how academic stress increases the risk of internet gaming addiction. This study clarifies the underlying mechanism through which academic stress affects internet gaming addiction: General strain theory (12) explains why academic stress elevates the risk of internet gaming addiction; Stress and coping theory (15) illustrates how anxiety transforms academic stress into internet gaming addiction as a maladaptive coping behavior; Ecological systems theory (22) identifies the social context in which this process is attenuated, namely the buffering effect exerted by high peer support. This integrated framework reveals both the psychological mechanism and boundary conditions linking stressors to behavioral outcomes, offering a more integrated theoretical explanation for this field.

### 4.5 Limitations and future directions

This study has several limitations. First, the data were collected solely through self-report questionnaires administered to college students. Such single-source, self-reported data are susceptible to social desirability bias and are inherently subjective, which may introduce common method bias and thus interfere with the statistical estimation of the relationships among variables. Future research could adopt a multiple-informant assessment approach by incorporating other-report data from teachers or peers, so as to enhance the objectivity of the measurements and the reliability of the research conclusions. Second, due to the cross-sectional research design, this study is unable to establish the temporal precedence among the variables, thereby making it difficult to confirm the direction of causality. Future research could adopt a longitudinal tracking design with data collection at multiple time points to further clarify the causal direction among the variables.Third, given that the sample was drawn solely from Chinese college students, it remains unclear whether the conclusions of this study can be generalized to other cultural backgrounds. Future research could conduct cross-cultural comparisons among broader cultural populations to examine the cultural stability and generalizability of these findings. Fourth, this study adopted a cross-sectional design, which precludes establishing the temporal sequence among academic stress, anxiety, and internet gaming addiction. Reverse causation is also plausible: for example, problematic gaming may lead to academic difficulties, which in turn increase academic stress and anxiety. Therefore, the observed associations should be interpreted as correlational rather than causal, and future research should employ longitudinal designs to clarify the direction of these relationships. Fifth, this study employed convenience sampling, with intact classes used as the unit of group administration. There may be intra-class homogeneity among students within the same class, which may lead to a design effect. Therefore, the sample may not fully represent the broader college student population, and the generalizability of the findings should be interpreted with caution.

### 4.6 Implications for practice

This study has important practical implications for the prevention and intervention of internet gaming addiction among college students. First, given that academic stress is a significant risk factor for internet gaming addiction among college students, schools can strengthen the establishment of a support system oriented toward student development, thereby alleviating students’ academic stress (33). Second, this study reveals that anxiety mediates the relationship between academic stress and internet gaming addiction; schools can offer specialized courses on emotional management to enhance college students’ emotion regulation skills and promptly relieve anxiety. Psychological counseling, such as strengths-based cognitive behavioral therapy (34), may also be provided to alleviate anxiety. Third, the findings indicate that peer support plays a protective role by weakening the indirect pathway through which academic stress affects internet gaming addiction via anxiety. Therefore, schools can promote peer support through organizing peer support groups and implementing peer tutoring programs, thereby reducing the risk of internet gaming addiction.

## 5 Conclusions

This study found that academic stress was significantly associated with internet gaming addiction among college students, with anxiety mediating this relationship and peer support moderating this indirect effect. Specifically, peer support exerted a stage-specific moderating effect by buffering only the first stage of the mediating pathway (academic stress → anxiety): the association between academic stress and anxiety was stronger for those with lower peer support, whereas it was weaker (yet still significant) for those with higher peer support. However, peer support did not moderate the direct effect of academic stress on internet gaming addiction or the second stage of the mediating pathway (anxiety → internet gaming addiction).

## Data Availability

The original contributions presented in the study are included in the article/supplementary material. Further inquiries can be directed to the corresponding author.
